# Comparison of functional and oncological outcomes of innovative “three-port” and traditional “four-port” laparoscopic radical prostatectomy in patients with prostate cancer

**DOI:** 10.1186/s12894-021-00787-7

**Published:** 2021-02-08

**Authors:** Ben Xu, Si-da Cheng, Yi-ji Peng, Qian Zhang

**Affiliations:** Department of Urology, National Urological Cancer Center, Peking University First Hospital and Institute of Urology, Peking University, 8 Xishiku Street, Xicheng District, Beijing, 100034 China

**Keywords:** Three-port, Laparoscopic radical prostatectomy, Prostate cancer, Continence, Oncological control

## Abstract

**Background:**

To compare the functional and oncological outcomes between innovative “three-port” and traditional “four-port” laparoscopic radical prostatectomy (LRP) in patients with prostate cancer (PCa).

**Methods:**

We retrospectively collected the data of PCa patients treated at our institutions from June 2012 to May 2016. According to the inclusion criteria, a total of 234 patients were included in the study, including 112 in group A (four-port) and 122 in group B (three-port). The perioperatively surgical characteristics, functional and oncological outcomes were compared between groups.

**Results:**

There were no statistical differences in the baseline parameters between these two groups. Compared with group A, the operative time (OT) and estimated blood loss (EBL) were significantly less in group B. On follow-up, the rate of positive surgical margin (PSM), prostate specific antigen (PSA) biochemical recurrence and continence after LRP did not show any statistically significant difference between the groups. An identical conclusion was also received in comparison of overall survival (OS) and biochemical recurrence-free survival (BRFS) between both groups.

**Conclusions:**

Innovative “three-port” LRP can significantly shorten the OT and reduce the EBL compared with the traditional “four-port” LRP. Meanwhile, it does not increase the rate of PSM and PSA biochemical recurrence. “Three-port” LRP could be popularized in the future in view of its superior surgical technique, considerably better functional outcomes and remarkable oncological control.

## Background

PCa is the second most common cancer in males (15% of newly diagnosed tumors), and the fifth major cause of cancer death worldwide [1]. The incidence of PCa is also increasing in China, which has become the highest incidence tumor in the male genitourinary system [2, 3]. For the localized PCa, RP is still the first-line treatment option, enabling patients to obtain more than 10 years of life expectancy [4].

Since LRP was reported in the 1990s [5], it has been widely applied based on its advantage of less surgical trauma and faster recovery. Moreover, with the emergence of robotic surgical platform, its unique three-dimensional vision, fine intracavitary manipulation and ergonomic design make robot-assisted laparoscopic radical prostatectomy (RARP) widely used throughout the world in the recent 10 years [6–8]. Although RARP can overcome the disadvantages of the traditional LRP and shorten the learning curve, it must be admitted that its cost is relatively high [9]. Such huge medical costs thus make it difficult to promote in the developing countries. To make a balance between a low price and more precise manipulation, our center innovatively adopted “three-port” LRP instead of the traditional “four-port” LRP from the year of 2012.

In this investigation, we retrospectively compared the perioperatively surgical parameters between the innovative “three-port” and traditional “four-port” LRP. More importantly, the functional and oncological outcomes including PSM, PSA biochemical recurrence, continence, short-term OS and BRFS were also compared to make this technique more practical and suitable for the future promotion.

## Methods

We retrospectively collected the data of PCa patients receiving LRP at our institutions from June 2012 to May 2016. The diagnosis of PCa was confirmed by a transrectal needle biopsy at our institutions 1–8 weeks before the LRP.

From the year of 2012, patients who met the following criteria were included into this trial: (1) All of the patients were confirmed by a transrectal needle biopsy with the assistance of magnetic resonance imaging preoperatively at our institutions; (2) The operation was performed between June 2012 and May 2016; (3) It was operated by a single surgeon (Dr. Qian Z) based on the previous medical records. The exclusion criteria included: (1) The medical record was fragmentary with incomplete perioperative characteristics [age, body mass index (BMI), prostate volume, PSA level, Gleason Score, OT, EBL, drainage indwelling days, hospitalization days, surgical complications, postoperatively pathological stages, and PSM]; (2) Patients who had undergone neoadjuvant androgen deprivation therapy; (3) Patients with preoperatively suspicious lymph node metastatic disease; (4) Patients with previously major abdominal surgery; (5) Patients with history of transurethral resection of the prostate; (6) Patients who received nerve sparing radical prostatectomy; (7) Salvaged LRP after radiation therapy. Patients who met the following criteria were operated by the “three-port” technique: (1) the volume of prostate measured by B-ultrasonography preoperatively was less than 40 ml; (2) B-ultrasonography showed that the middle lobe of prostate did not protrude into the bladder; (3) the BMI was less than 24 kg/m^2^. The other patients who were not in conformity with the above requirements were still performed by the “four-port” LRP.

Accordingly, a total of 234 patients were included in the study, including 112 in group A (four-port) and 122 in group B (three-port). Among them, a total of 17 “three-port” patients were intraoperatively converted to “four-port”. Five cases had intra-operative bleeding requiring additional suction port while in other 12 the prostate was found to be large and hence needed an additional port for proper exposure and dissection.

The perioperatively surgical characteristics, functional and oncological outcomes were compared between groups. The surgical characteristics included OT, EBL, hospitalization and drainage days. The recovery of urinary control function after operation was evaluated according to the use of daily urine pad, and 0–1 piece of urinary pad per day was defined as a satisfactory urinary continence [10]. The oncological outcomes were evaluated by the parameters of PSM, PSA biochemical recurrence rate, OS and BRFS obtained by the follow-up records. The PSA biochemical recurrence was defined as PSA ≥ 0.2 ng/ml, OS was defined as the time from the end of operation to death or follow-up due to any reason, and BRFS was defined as the time from the end of operation to the occurrence of PSA biochemical recurrence or death or the end of follow-up.

All the data were analyzed by SPSS 20.0 versions. Categorical variables were represented by frequency and percentage. Continuous variables conforming to normal distribution were represented by mean ± standard deviation (SD), and continuous variables not conforming to normal distribution were represented by median and range. Chi-square test or Fisher exact test were used for categorical variables, with t test or Mann–whitney test for continuous variables. Bilateral *P* < 0.05 was defined as a statistically significant difference.

## Results

The baseline characteristics of patients in both groups were listed in Table 1. There were none of statistical differences in the baseline parameters between the two groups. The perioperatively surgical data of these two groups were shown in Table 2. The average OT in group A was 105.06 min, and that of the group B was 92.28 min (*P* = 0.001). The average EBL in group A was 121.90 ml, and that of the group B was 103.85 ml (*P* = 0.031). The postoperative hospitalization in group A was 4.54 days, and that of the group B was 4.50 days (*P* = 0.812). The drainage days in group A was 3.47 days, and that of the group B was 3.43 days (*P* = 0.743). Compared with group A, the OT and EBL were significantly less in group B.

**Table 1:** The patients’ baseline characteristics in both groups

	Overall	Group_A	Group_B	*P* value
Cases	234	112	122	
Age [mean (SD)]	66.92 (7.80)	67.07 (8.75)	66.79 (6.85)	0.781
TPSA [mean (SD)]	13.90 (6.43)	14.22 (6.24)	13.60 (6.62)	0.465
Biopsy Gleason scores (%)				0.597
3 + 3	69 (29.5)	29 (25.9)	40 (32.8)	
3 + 4	49 (20.9)	28 (25.0)	21 (17.2)	
3 + 5	14 (6.0)	5 (4.5)	9 (7.4)	
4 + 3	85 (36.3)	41 (36.6)	44 (36.1)	
4 + 4	6 (2.6)	3 (2.7)	3 (2.5)	
4 + 5	11 (4.7)	6 (5.4)	5 (4.1)	
cT stage (%)				0.638
cT2a	11 (4.7)	4 (3.6)	7 (5.7)	
cT2b	32 (13.7)	14 (12.5)	18 (14.8)	
cT2c	160 (68.4)	81 (72.3)	79 (64.8)	
cT3a	28 (12.0)	11 (9.8)	17 (13.9)	
cT3b	3 (1.3)	2 (1.8)	1 (0.8)

**Table 2:** Intraoperatively surgical parameters in both groups

	Overall	Group A	Group B	*P* value
Cases	234	112	122	
OT [mean (SD)]	98.40 (30.27)	105.06 (30.79)	92.28 (28.57)	0.001
EBL [mean (SD)]	112.49 (64.05)	121.90 (67.09)	103.85 (60.11)	0.031
Hospitalization [mean (SD)]	4.52 (1.14)	4.54 (1.34)	4.50 (0.93)	0.812
Drainage days [mean (SD)]	3.45 (0.90)	3.47 (1.01)	3.43 (0.79)	0.743

The functional and oncological outcomes of the two groups were revealed in Table 3. There was no significant difference in the postoperative T stage and Gleason scores. By analyzing the oncological outcomes of two methods, the mean rate of PSM in the two groups was 32.1% and 32.0% respectively (*P* = 1.000), and the PSA biochemical recurrence rate was 10.7% and 10.7% respectively (*P* = 1.000). In the evaluation of the degree of urinary control recovery at 3 months, 6 months and 12 months after operation, the satisfied urinary continence in group A was obtained in 92/112 cases (82.1%), 97/112 cases (86.6%) and 102/112 cases (91.1%). The corresponding rate was 94/122 cases (77.0%), 99/122 cases (81.1%) and 104/122 cases (85.2%) in group B. The *P* value for each period between both groups were 0.423, 0.340 and 0.242 respectively. Above all, the similar results were obtained in the rate of PSM, PSA biochemical recurrence and continence after “three-port” or “four-port” LRP without any statistically significant difference (*P* > 0.05).

**Table 3:** Patient characteristics

	Overall	Group_A	Group_B	*P*
Cases	234	112	122	
pT_stage (%)				0.809
pT2b	16 (6.8)	8 (7.1)	8 (6.6)	
pT2c	125 (53.4)	63 (56.2)	62 (50.8)	
pT3a	56 (23.9)	24 (21.4)	32 (26.2)	
pT3b	37 (15.8)	17 (15.2)	20 (16.4)	
Patho_GS (%)				0.638
3 + 3	16 (6.8)	7 (6.2)	9 (7.4)	
3 + 4	70 (29.9)	27 (24.1)	43 (35.2)	
4 + 3	100 (42.7)	57 (50.9)	43 (35.2)	
4 + 4	3 (1.3)	1 (0.9)	2 (1.6)	
4 + 5	30 (12.8)	12 (10.7)	18 (14.8)	
5 + 3	7 (3.0)	3 (2.7)	4 (3.3)	
5 + 4	8 (3.4)	5 (4.5)	3 (2.5)	
SurgicalMargin (%)				1
Negative	159 (67.9)	76 (67.9)	83 (68.0)	
Postive	75 (32.1)	36 (32.1)	39 (32.0)	
BioChemicalRecurrence (%)				1
Negative	209 (89.3)	100 (89.3)	109 (89.3)	
Positive	25 (10.7)	12 (10.7)	13 (10.7)	
Continence (%)				
Third month	186 (79.5)	92 (82.1)	94 (77.0)	0.423
Sixth month	196 (83.8)	97 (86.6)	99 (81.1)	0.340
Twelfth month	206 (88.0)	102 (91.1)	104 (85.2)	0.242

**Fig. 1:**
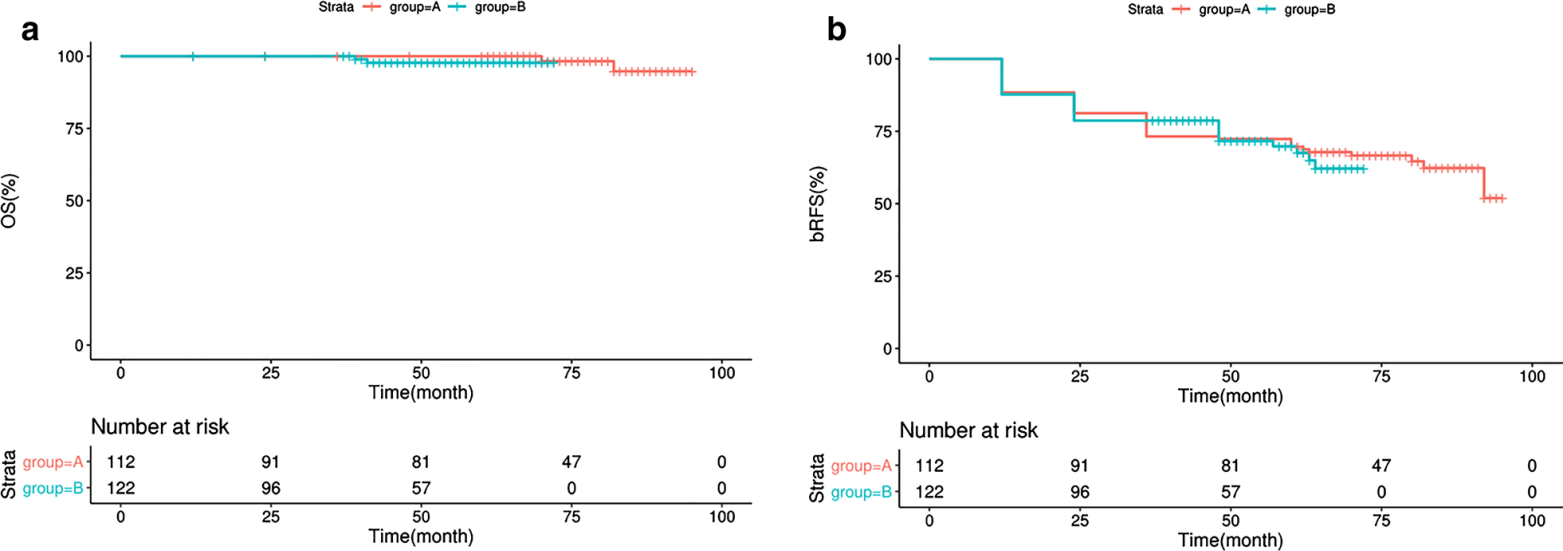
The survival rate of PCa patients after LRP. **a** The OS rate of PCa patients in group A (red line) and group B (green line). **b** The BRFS rata of PCa patients in group A (red line) and group B (green line)

By depicting a curve of OS and BRFS, a more accurate reflection of oncological outcomes was demonstrated in Fig. 1. In the follow-up periods, the OS was 100% with the 71.6% of BRFS in group A, and the OS was 100% with the 73.2% of BRFS in group B. There was no significant difference in OS (*P* = 0.300) and BRFS (*P* = 0.800) between these two groups.

## Discussion

For the early localized PCa, RP is still the most widely used treatment method. With the continuous progress of technology, LRP and RARP both embody the advantages of small trauma, less postoperative pain and rapid postoperative recovery. What’s more, RARP has unique advantages such as flexible operation equipment, three-dimensional vision and short learning curve, which has been widely applied by many centers in many developed countries [11]. Several systematic reviews and meta-analysis results had shown that RARP took the advantages of perioperatively surgical data, lower PSM, satisfied urinary continence and sexual function over LRP, but some researchers believed that there existed no dramatic difference in tumor prognosis between LRP and RARP [12–15]. However, it is worth noting that many studies have shown that RARP has a higher cost than LRP due to the increased cost of surgical instruments [9, 14, 16]. Based on the limitation of cost and medical resources, LRP is still an important choice for many underdeveloped and developing countries.

Since it was first reported in 1997 [17], the traditional “four-port” or “five-port” LRP has been widely used in the world. Reviewing the literatures concerning traditional LRP, Rassweiler et al. [18] reported 219 cases with an average OT of 218 min and an average EBL of 800 ml; Hu et al. [19] reported 358 cases with an average OT of 246 min and an average EBL of 200 ml; Ploussard et al. [20] reported 219 cases with an average OT of 175.5 min and an average EBL of 800 ml. Compared with the results above and the traditional “four-port” LRP performed by ourselves, our clinical practice in “three-port” LRP showed that the average OT (92.28 min) and EBL (103.85 ml) were notably improved. It is believed that the improvement of perioperatively surgical data is mainly related to the rationality and advantages of the “three-port” technique. Additionally, it may also be contributed to the fact that the surgeon launched the innovative “three-port” LRP after fully mastering the traditional “four-port” LRP. Retrospectively analyzing our database, nearly 100 cases of traditional “four-port” LRP were completed before the “three-port” LRP was developed in 2012. However, with the progress of current technology and the visualization of laparoscopic teaching methods, we believe that young doctors can directly perform the “three-port” LRP even though the youths are still in the initial stage of the traditional “four-port” LRP without spending the learning curve. It is not necessary to go through the learning curve process from “four-port” to “three-port” technique. Certainly, it is strongly suggested that the young doctors should perform the “three-port” LRP with the guidance of experienced doctors who have been skilled and proficient in this technique.

PSM is an important index to evaluate the prognosis of PCa after RP, which is closely related to PSA biochemical recurrence and postoperative adjuvant treatment [21]. According to the systematic review, it is uncertain whether RARP has advantages over LRP in controlling the PSM. It has been reported that the rate of PSM in LRP is 12.0–22.2%, while that of RARP is 13.5–22.5% [12, 19, 22, 23]. Our results suggest that the rate of PSM in “three-port” and “four-port” technique were 32.0% and 32.1% without any statistical difference. BCR is another critical index of oncological outcomes closely related to PSM. Our conclusion revealed that the 1-year BCR of “three-port” and “four-port” was both 10.7% similarly compared with the recent literatures [24, 25]. The above results indicated that “three-port” LRP did not significantly increase PSM and BCR, which could be controlled at a better level on the basis of rich surgical experience. Finally analyzed by the description of OS and BRFS, it was further confirmed that “three-port” LRP could guarantee a satisfactory survival rate.

The recovery of urinary control is a considerable aspect to evaluate the functional prognosis after RP. Asimakopoulos et al. [25] reported that the urinary control rates at 3 months, 6 months and 1 year after LRP were 63.3%, 75.0% and 83.3%, respectively. Ploussard et al. [20] reported that the urinary control rates of 1377 patients with LRP at 3 months, 6 months and 1 year were 39.4%, 58.9% and 68.5%, respectively. Porpiglia et al. [26] reported that the urinary control rates of LRP at 3 months, 6 months and 1 year were 61.6%, 73.3% and 83.3%, respectively. Our results stated that the urinary control rates of “three-port” LRP at 3 months, 6 months and 1 year were 77.0%, 81.1% and 85.2% respectively, providing a stable recovery of urinary continence without increasing the incidence of postoperative urinary incontinence.

In conclusion, the “three-port” LRP can significantly shorten the OT and EBL, without increasing the rate of PSM, PSA biochemical recurrence and urinary incontinence. More importantly, it could obtain a considerable outcome in the tumor prognosis. Its main advantages were: (1) more fast recovery by reducing a puncture trocar; (2) avoiding improper traction and auxiliary operation by inexperienced assistants; (3) a triangle layout providing sufficient space, meanwhile reducing the fatigue of the surgeon. Admittedly, it must be addressed that there still existed some defects and disadvantages in the “three-port” LRP: (1) it is mainly suitable for extraperitoneal operation, not for the transperitoneal manipulation. If the extended lymph node dissection plans to be implemented, it needs to be converted to the traditional “four-port” LRP; (2) if the prostate volume is larger, the operator will be limited in a narrow space, and thus the assistant is required to assist in providing an adequate exposure by adding an additional port; (3) if there is much bleeding during the operation, the assistant is still demanded to suck the blood by using an aspirator in the fourth port.

This study was a retrospectively and non-randomized controlled study with inherent selection bias. In this study, the follow-up time of tumor prognosis and functional prognosis is relatively short. In the future, a prospectively randomized controlled study with a large sample and long-term follow-up is needed to further confirm the advantages of “three-port” LRP in functional and oncological outcomes.

## Conclusions

In conclusion, innovative “three-port” LRP can significantly shorten the OT and reduce the EBL compared with the traditional “four-port” LRP. Meanwhile, it does not increase the rate of PSM and PSA biochemical recurrence. “Three-port” LRP could be popularized in the future in view of its superior surgical technique, considerably better functional outcomes and remarkable oncological control.

## Data Availability

The datasets analysed during the current study available from the corresponding author on reasonable request.

## Abbreviations

LRP: Laparoscopic radical prostatectomy
PCa: Prostate cancer
OT: Operative time
EBL: Estimated blood loss
PSM: Positive surgical margin
PSA: Prostate specific antigen
OS: Overall survival
BRFS: Biochemical recurrence-free survival
RARP: Robot-assisted laparoscopic radical prostatectomy
BMI: Body mass index
DVC: Dorsal vein complex
SD: Standard deviation
